# Temporal Evolutionary Dynamics of Norovirus GII.4 Variants in China between 2004 and 2015

**DOI:** 10.1371/journal.pone.0163166

**Published:** 2016-09-20

**Authors:** Niu Qiao, Xuan-Yi Wang, Lei Liu

**Affiliations:** 1 Shanghai Public Health Clinical Center, Fudan University, Shanghai, P. R. China; 2 Institutes of Biomedical Sciences, Fudan University, Shanghai 200032, P. R. China; 3 Key Laboratory of Medical Molecular Virology, MoE/MoH, Fudan University, Shanghai 200032, P. R. China; National Institute of Health, ITALY

## Abstract

**Background:**

Noroviruses are one of the major causes of acute human nonbacterial gastroenteritis, and genotype II.4 (GII.4) has accounted for the majority of adult outbreaks. In addition, novel epidemic strain emerges every 2 to 3 years and spreads globally in months. There are little data reporting the evolutionary dynamics of GII.4 variants along a specific period in China.

**Methods:**

All norovirus GII.4 sequences in China were downloaded from GenBank Database. Phylogenetic tree was constructed by MEGA and Bayesian evolutionary inference techniques were applied by BEAST software to study the genetic relationships, evolution rate, evolutionary time scale and the demographic history of GII.4 variants. Homology models were predicted by SWISS-MODEL to analyze the spatial structure changes.

**Results:**

During the 12-year period, 624 GII.4 sequences were subtyped into six GII.4 variants (clusters). A rate of 4.74×10^−3^, 6.99×10^−3^ and 7.68×10^−3^ nucleotide substitutions/site/year for the strict, uncorrelated log-normal and uncorrelated exponential derivation clocks were estimated, respectively. Three amino acid mutations (G255S, S393G and H414P) in both Den Haag_2006b sub-clusters and six mutations (I244T, N309S, A377T, T244I, T377A and S393G) in three Sydney_2012 sub-clusters were observed.

**Conclusions:**

The temporal distribution pattern of noroviruses GII.4 lineages in China was similar to the worldwide observation. The evolutionary rate of GII.4 was consistent with the global studies. Amino acid changes in the vicinity of norovirus epitope may have profound influences on carbohydrate binding affinity between different sub-clusters of norovirus variants. Hence understanding the evolutionary dynamics of norovirus is of great value for developing effective prevention and control strategies.

## Introduction

Norovirus (NoV) is the most common causative agent of acute nonbacterial gastroenteritis in all ages, and almost 267 million NoV infections and over 200,000 deaths, mostly in infants and the elderly, have been identified yearly worldwide[1–2]. These viruses cause gastrointestinal disease, resulting in recurrent bouts of vomiting and diarrhea that typically last 24–48 hours, although virus shedding can be prolonged for several weeks [3]. NoV is of concern given the significant burden it places on public health, particularly hospitals and aged-care facilities [4]. A thorough understanding of the evolutionary pattern, evolutionary rate, genetic diversity and epidemic cycles of NoV may help interpret how these viruses change, evade the host immunity and adapt to hosts, which is important to develop strategies such as antiviral drugs and vaccines against NoV.

NoV contains a linear, single-stranded, positive-sense polyadenylated RNA genome of approximately 7.7 kb which contains three open reading frames (ORFs) [5]. ORF2 and ORF3 encoded a major structural protein (VP1) and a minor capsid protein (VP2), respectively [5–6]. Genetically, according to the genetic characteristics, this virus is classified into six genogroups (GI-GVI). These genogroups are further divided into ~40 genotypes based on viral protein 1 (VP1) sequences [7]. Two major genogroups, GI and GII, mainly cause human acute gastroenteritis [8]. Multiple NoV genotypes have been found cocirculating in human populations in which the GII genotype 4 (GII.4) has been the predominant genotype that cause over 80% of all NoV infections [3–4]. It is also the major circulating genotype in community and health-care settings with periodic emergence of novel GII.4 variants every 2–3 years with a total of six major GII.4 NoV pandemics reported since 1995: the US 95/96 (1996)[9], Farmington Hills (2002)[10], Hunter (2004)[11], Den Haag (2006b)[12], New Orleans (2009)[13] and Sydney 2012 (2012)[14]. In addition, other GII.4 variants have also been described, including Asia 2003[15], Yerseke 2006a [16], Osaka 2007[17], and Apeldoorn 2008 [18] although they caused only localized epidemics in specific geographical regions [15–19]. It is believed that the evolution of NoV GII.4 is similar to that of influenza viruses with constant change of host specificity by accumulation of point mutations and genomic recombination under selection of the host immune surveillance [20].

Recent advances of genetic analysis algorithms enable us to obtain the evolutionary information of various viruses. In this study, we aimed to understand the distribution patterns together with the genetic diversity of GII.4 NoV variants in China through time. Phylogenetic relationship of GII.4 NoV proteins was analyzed by maximum likelihood approach, using sequences from strains with known detection dates and locations collected in the country from 2004 to 2015. Evolutionary time scale and population dynamics of viral genes were estimated by a Bayesian coalescent approach using Markov chain Monte Carlo (MCMC) method. In addition, capsid protein structural modeling was conducted to investigate the potential microevolution mechanism for viral population expansions of a specific variant.

## Materials and Methods

### Data Collection

In order to obtain all available Chinese genetic data sets representative of the GII.4 noroviruses, we retrieved the gene sequences by searching the corresponding taxonomy ID of the GII.4 noroviruses (Taxonomy ID: 489821) from human host together with a keyword “China” in NCBI’s GenBank Database[21] (accessed on March 9, 2016). The corresponding searching key was “txid489821 [Organism:exp] AND China”. The sampling time and location of isolation for each of the NoV isolates were retrieved from GenBank or the publication associated with the sequence, and the year of isolation was used to calculate evolutionary rate. In order to facilitate the downstream analysis, we renamed all the sequence in GenBank ID_Location_Year format. Sequences containing the above information were reserved to maintain enough phylogeographic information and to ensure the accuracy of final results when doing subsequent analyses.

### Sequence Analyses

Of all the 1034 Chinese NoV GII.4 sequences downloaded from GenBank, 888 sequences (S1 Table) containing sampling time and location were chosen for the study and were also genotyped using the NoV automated online genotyping tool offered by the Netherlands National Institute for Public Health and the Environment (RIVM) spanned the continuous genomic region covering regions A, B, C, D, and E [22]. All sequences analyzed in this study were aligned using the multiple sequence alignment tool MUSCLE [23] in the Molecular Evolutionary Genetics Analysis (MEGA) software version 6.06 [24] with default parameters values and manual editing was carried out to truncate the sequences at both the 5’ and 3’ ends. For the full length VP1 sequences set, the total alignment sequence length was 7606, and we retrieved sequences from site 5084 to 6707, resulting in a result of 1623nt. For the subset of 526 partial sequences from region C, we retrieved sequences from site 5084 to 5365, resulting in a length of 281nt.

### Phylogenetic Analyses

To evaluate the evolutionary relationships among NoV GII.4 variants in China, a phylogenetic tree was inferred by Maximum Likelihood (ML) reconstruction based on the nucleotide alignment of the full-length ORF2 sequences as implemented in the MEGA software v6.06 [24]. A heuristic tree search was performed using the Nearest-Neighbor Interchange (NNI) algorithm, and a Neighbor-Joining (NJ) tree was chosen as initial tree. A separate Hasegawa-Kishino-Yano (HKY [25]) substitution model with gamma-distributed rate variation among sites [26] was applied and the complete deletion parameter for gaps/missing data treatment [27] was set for the calculation. The statistical significance of phylogenies constructed was estimated by bootstrap method with 1000 replicates. The tree was also displayed with the MEGA software v6.06 [24]. Additionally, approximate maximum likelihood tree was also constructed by TREE-PUZZLE v5.3 program[28]. Support for the nodes was obtained by quartet puzzling analysis (QP) performed in TREE-PUZZLE with the Gamma distribution and 10,000 replicates. Both the above two methods gave similar tree topologies (S2 Fig)

### Evolutionary and Demographic Analyses of NoV GII.4 Variants

The parameter values for the best-fit model of nucleotide substitutions were determined using Akaike Information Criterion (AIC) as implemented in jModelTest v2.1.10 program [29]. The rate of nucleotide substitution per site, the time to the most recent common ancestor (TMRCA), and the demographic history of NoV GII.4 were jointly estimated using the Bayesian Markov chain Monte Carlo (MCMC) approach as implemented in the BEAST software v1.8.2 package [30]. The alignment was created and edited as described above, and the corresponding dates were assigned using the BEAUTi application [30], part of the BEAST package. The best-fit nucleotide model of substitution (S1 File) was Hasegawa-Kishino-Yano (HKY [25]) with gamma-distributed rate variation among sites (HKY+I+G) [26]. A flexible demographic tree prior named GMRF Bayesian Skyride was selected to estimate the NoV GII.4 evolutionary rate. Since no pre-specified parametric demographic model is available from NoV GII.4 Den Haag_2006b variant showing cyclic annual/seasonal behavior in China, the Bayesian Skyline model [31] for population growth was selected to estimate the variant effective population size through time.

The MCMC analysis was performed three times each for 100 million generations, sampling every 10000^th^ generation and removed 10% as chain burn-in, either assuming a constant rate of evolution across the tree (strict molecular clock), or using relaxed-clock models (lognormal and exponential), which model a molecular rate that varies among lineages[32].

In all cases, statistical uncertainty in the parameter estimates across the sampled trees was reflected in the 95% highest probability density (HPD) intervals, and the convergence of continuous parameters was assessed by calculating the Effective Sample Size (ESS) (greater than 200) using the TRACER v1.6 program (http://tree.bio.ed.ac.uk/software/tracer/) after excluding the initial 10% of the run. The programs TreeAnnotator v1.8.2 and FigTree v1.8.0, parts of BEAST package [30], were used to summarize the posterior tree distribution data produced by BEAST into a Maximum Clade Credibility (MCC) tree, where the branch length was calibrated to reflect temporal patterns and to visualize the annotated tree, respectively.

### Homologous modeling of the NoV GII.4 Den Haag_2006b and Sydney_2012 Sub-lineages

The consensus (majority) amino acid sequences of the two NoV GII.4 Den Haag_2006b sub-lineages and three Sydney_2012 sub-clusters were determined using MEGA software v6.06 [24]. Three-dimensional structure of the P domain was constructed by the homologous modeling database SWISS-MODEL (http://swissmodel.expasy.org/) using the crystal structures of P domain of Norovirus VA387 (PDB accession code: 2OBR [33]) as the template. Residue changes between the two clusters were mapped to the model by PyMol v1.3 software. Residues were numbered according to the reference norovirus strain (GenBank accession No: ABV55634.1).

## Results

### Temporal Distribution of NoV GII.4 Variants in China

**Fig 1:**
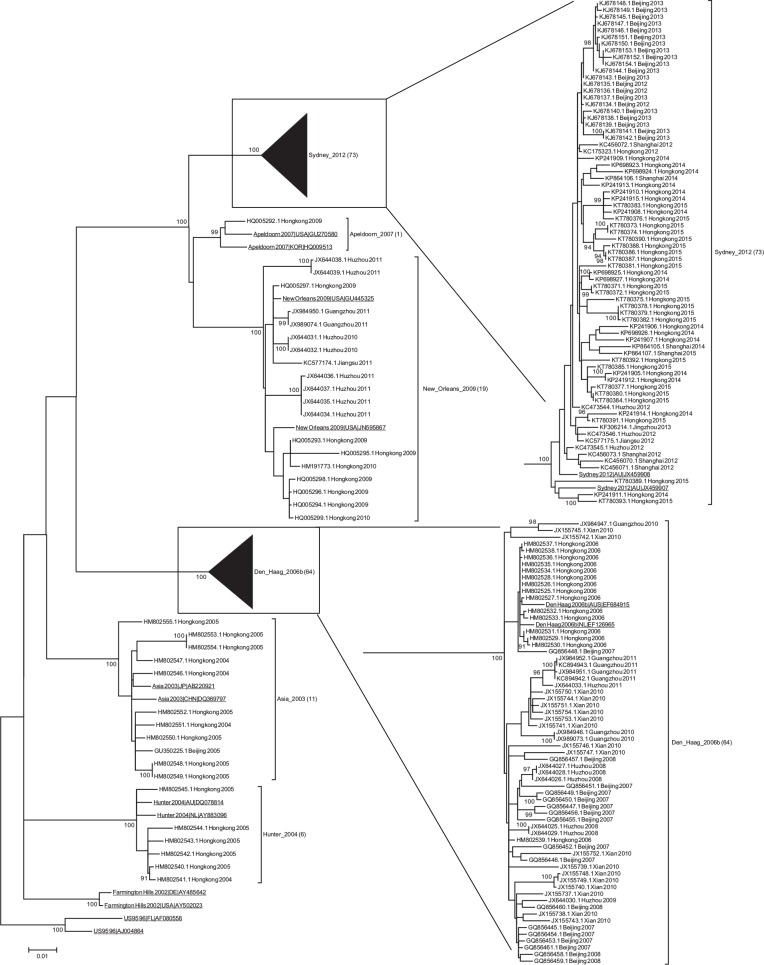
Phylogenetic Tree of GII.4 NoV variants in China. Phylogenetic relationships of full-length capsid GII.4 strains sequences, constructed by MEGA 6.06 software (174 Chinese sequences and 16 reference sequences, 1623 nt, HKY+I+G4 [symmetric, with allowance for invariant sites and gamma substitution rate variation], 1000 bootstraps). Sequence name was formatted as follows: GenBank accession number_location_year. Reference strains (first complete capsid sequences submitted to the public databases) are underlined. The model was selected using jModelTest v2.1.10 program. The number of sequence variants is listed in parentheses. The tree is drawn to scale; the scale bar labeled 0.01 indicates the average distance by nucleotide differences (as a percentage). The percentage bootstrap values in which the major groupings were observed among 1000 replicates are indicated. Bootstrap values above 900 are shown as a percentage.

A total of 647 sequences were confirmed as NoV GII.4, 624 of which were subtyped into NoV GII.4 ORF2 variants, and 23 of the 647 sequences could not be assigned to known GII.4 ORF2 variants and were excluded from further analysis. 174 full capsid sequences collected in our study with 8 reference NoV GII.4 variant strains (n = 16, each strain contains two sequences), and a subset of 526 partial sequences from region C were phylogenetically analyzed (Fig 1 and S1 and S2 Figs). The aligned complete capsid gene nucleotide sequences correspond to positions 5084 and 6707 (1623 nt) in the genome of Hu/NLV/Hawaii virus/1971/US (GenBank accession No. U07611). The detailed data are shown S1 Table.

The ML phylogenetic analysis of 174 full-length ORF2 sequences and 526 region C sequences demonstrated the presence of six variants in different provinces of the country during the period of 2004–2015 (Fig 1 and S1 and S2 Figs). The most prevalent variants were Den Haag_2006b in 2006–2011, New Orleans_2009 in 2009–2011, and Sydney_2012 in 2012–2015; the three minor variants were Asia_2003 in 2004–2005, Hunter_2004 in 2004–2005 and Apeldoorn_2007 in 2009 (Table 1 and S3 Fig). Clustering into variants was consistent, as illustrated for region C sequences in S2 Fig. For most of the years analyzed, it was possible to detect at least two different variants co-circulating in China (Table 1 and S3 Fig), and a clear variation in the frequency of the different variants over time was also observed. In addition, the variants Den Haag_2006b, New Orleans_2009, and Sydney_2012 were detected in more than nine Chinese cities and the minor variants were detected at minor geographic locations with low frequencies (S2 Table).

**Table 1:** Distribution of the GII.4 NoV variants detected in China during the period 2004–2015 (n = 624)^a^.

GII.4 Variant	Year												Total
	2004	2005	2006	2007	2008	2009	2010	2011	2012	2013	2014	2015	
Asia_2003	3	8	1										12
Hunter_2004	1	5	1										7
Den_Haag_2006b			51	35	46	14	108	58	6	11			329
Apeldoorn_2007						1							1
New_Orleans_2009						12	10	26	1				49
Sydney_2012									65	98	33	30	226

^a^Blank indicates that there is no corresponding variant in that year.

### Evolutionary History of NoV GII.4 Variants

To reconstruct the time-scale of the NoV GII.4 epidemics in China, we performed a Bayesian coalescent analysis of the 174 full-length VP1 sequences between 2004 and 2015. Three different molecular clock models were used, which were a strict-clock, an uncorrelated log-normal model (UCLN), and an uncorrelated exponential derivation model (UCED). The most conservative clock, the strict model, estimated that NoV GII.4 VP1 evolved at a rate of 4.74×10^−3^ nucleotide substitutions/site/year (Table 2). The relaxed-clock estimations calculated higher rates of evolution, 6.99×10^−3^ and 7.68×10^−3^ nucleotide substitutions/site/year for the UCLN and UCED clocks, respectively. The same Bayesian approach was used to estimate the time to the most recent common ancestor (TMRCA) using each clock model. The mean age of the GII.4 VP1 most recent ancestor derived from the population analyzed was 19.6 years (19.7 to 21.4) by the strict clock, which would date the most recent ancestor to 1978, while the relaxed-clock models dated the ancestor to 2000.6 to 2000.8 (Table 2).

**Table 2:** Nucleotide substitution rates and divergence times over 12 years for the VP1 gene of GII.4 NoV variants in China(n = 174, 1623 nt)^a^.

Molecular clock	Nucleotide substitution rate	TMRCA by:	
	(10^−3^ substitutions/site/year)	No. of year	Date (range)
Strict clock	4.74 (4.12, 5.41)	19.6 (17.9, 21.4)	1994.4 (1978.4, 1996.1)
UCLN	6.99 (5.54, 8.59)	13.4 (12.4, 14.3)	2000.6 (1999.7, 2001.6)
UCED	7.68 (6.14, 9.29)	13.2 (12.4, 14.1)	2000.8 (1999.9, 2001.6)

^a^The nucleotide substitution rate is the mean rate for the three individual determinations. UCLN, uncorrelated log-normal clock. UCED, uncorrelated exponential deviation clock. TMRCA, time to most recent common ancestor. Values in parentheses are 95% HPDs.

**Fig 2:**
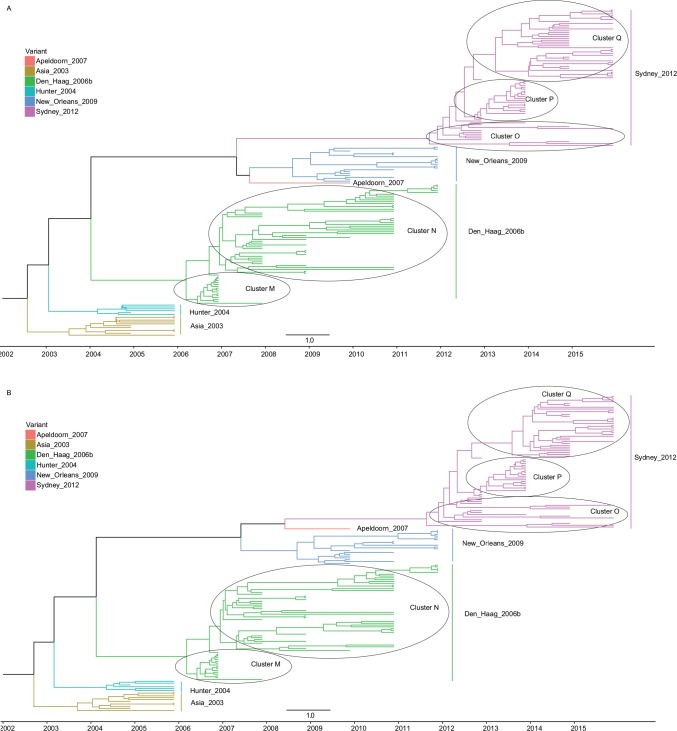
MCC tree of NoV GII.4 complete capsid gene (ORF2) sequences from China (n = 174, 1623 nt, HKY+I+G4). (A) MCC tree estimated by uncorrelated log-normal clock (UCLN). (B) MCC tree estimated by uncorrelated exponential deviation clock (UCED).

From the MCC trees (Fig 2A and 2B), we can see that there were two distinct sub-lineages in Den Haag_2006b variant: the ‘‘N” sub-lineage that emerged early 2007 is a descendant from the ‘‘M” sub-lineage. The identification of a few samples from Beijing (n = 1) and Hong Kong (n = 15) were grouped in sub-cluster “M”. In addition, samples from Beijing (n = 16), Guangzhou (n = 7), Huzhou (n = 7) and Xi’an (n = 18) were grouped within sub-cluster “N”. As for Sydney_2012 variant, there were three distinct sub-lineages: the early sub-cluster “O” was composed of samples from Shanghai (n = 4), Huzhou (n = 3) Jingzhou (n = 1), Jiangsu (n = 1) and Hong Kong(n = 5). The later sub-cluster “P” was entirely composed of 21 sequences from Beijing. Besides, the final sub-cluster was composed of sequences form Hong Kong (n = 35) and Shanghai (n = 3) (Fig 2A).

### Demographic History of the NoV GII.4 Den Haag_2006b and Sydney_2012 Variants

**Fig 3:**
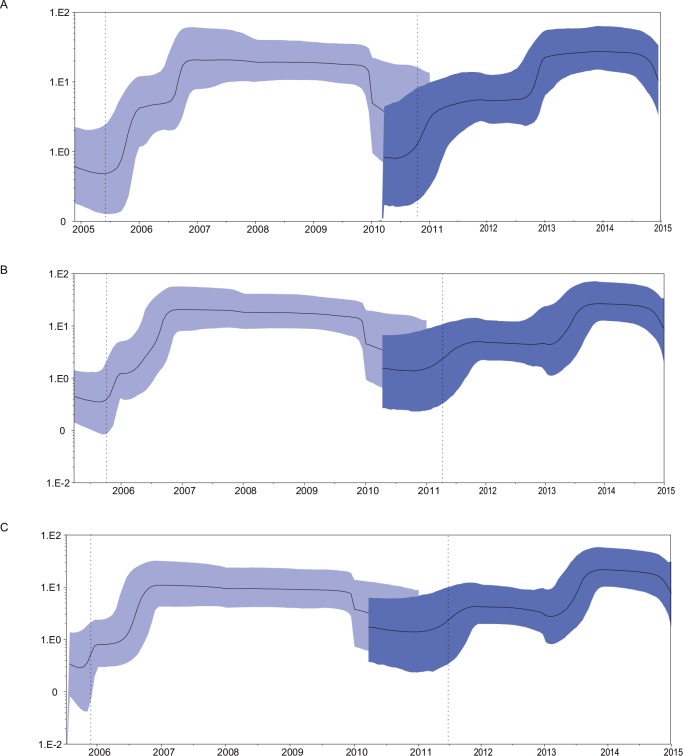
Bayesian skyline plot of the VP1 sequences of Den Haag_2006b variant (n = 64, lightblue) and Sydney_2012 variant (n = 73, darkblue). The Bayesian skyline plot was estimated under the HKY+I+G4 model. The MCMC chains were run for 100,000,000 steps. The Y-axis represents the effective population size on a logarithmic scale and the X-axis represents generation time (year). The solid black line represents the median posterior value over time. The 95% Highest Probability Density (HPD) intervals are shown as a shaded blue area. The lower value of the HPD95% interval and the mean value for the TMRCA are shown as a vertical dotted line. (A) Bayesian skyline plot estimated by strict molecular clock. (B) Bayesian skyline plot estimated by uncorrelated log-normal clock (UCLN). (C) Bayesian skyline plot estimated by uncorrelated exponential deviation clock (UCED).

We estimated of the demographic history of the NoV GII.4 Den Haag_2006b and Sydney_2012 lineages using Bayesian skyline plot (BSP) analysis. As a result, the Den Haag_2006b strain experienced a rapid expansion in late 2005 after which the population reached a maximum effective population size in mid to late 2006 (Fig 3A, 3B and 3C). In addition, a relatively constant effective population size values over 100 was seen during 2007 and 2010, but thereafter the values tended to be low (Fig 3A, 3B and 3C). However, the Sydney_2012 variant showed a different demographic pattern. The Sydney_2012 strain first experienced a relatively small fixed population size less than 10 during 2012 and 2013. Then, after a rapid expansion for almost one year (form 2013 to 2014), the population reached a maximum effective population size about 100 during 2014 and to date (2015) (Fig 3A, 3B and 3C).

### Structural Modeling the P domains of the NoV GII.4 Den Haag_2006b Sub-lineages

**Fig 4:**
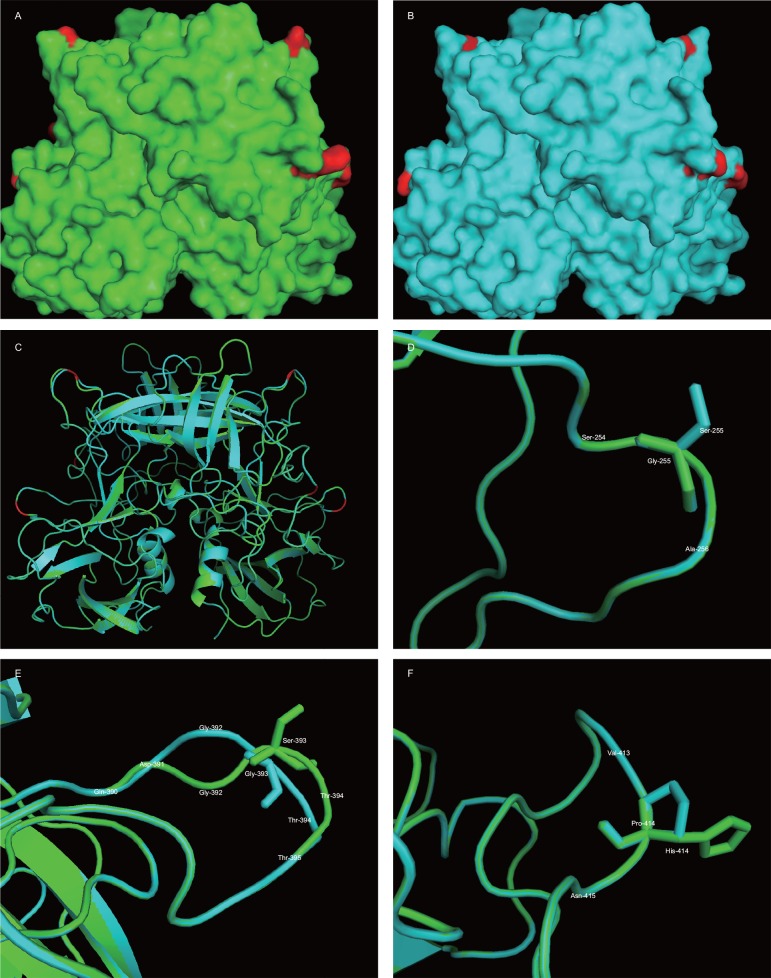
Structure modeling the P domain of the two NoV GII.4 Den Haag_2006b sub-lineages. Homology models for the P domain of sub-cluster “M” and sub-cluster “N” were generated using the crystal structures of P domain of Norovirus VA387 (PDB accession code: 2OBR) as the template. (A) Surface representation of the consensus sequence (GeneBank accession ID: HM802525.1) of sub-cluster “M” P dimer. (B) Surface representation of the sub-cluster (GeneBank accession ID: JX644030.1) “N” P dimer. (C) A superimposition of the two minimized ribbon structures predicted for sub-cluster “M” (green) and sub-cluster “N” (cyan). The positions of 255 (Gly-to-Ser), 393 (Ser-to-Gly) and 414 (His-to-Pro) are marked with red cartoon. (D) Cartoon structures representation of the subtle changes (Gly-to-Ser) at position 255. (E) The changes from Ser in sub-cluster “M” to Gly in sub-cluster “N” at position 393. (F) A mutation from His to Pro at position 414.

Multiple alignment of the corresponding protein sequences of Den Haag_2006b variant (n = 64) showed that sub-cluster ‘‘N” displayed a large number of substitutions (≥5 residues changes) at residues 255, 393, 407, 411, 412, 413, 414 and 425 in relation to sub-cluster ‘‘M” (S3 Table). Homology models for the P domain of the capsid dimer were predicted on each representative consensus strain, and changes that showed the differences of each cluster were highlighted on the superimposed structures (Fig 4). Three amino acid differences were observed between sub-cluster “M” and sub-cluster “N” consensus sequences, these included mutations, from sub-cluster “M” to sub-cluster “N”, at residues 255 (Gly-to-Ser) and 393 (Ser-to-Gly) and 414 (His-to-Pro). The residue 255 is located in the P1 domain of the major capsid protein (VP1) [19], and the residues 393 and 414 are located in epitope “D” and in the vicinity of (1 residue adjacent to antigenic epitopes) epitope “E” of the virus capsid [19, 34]. Our results suggest that these sites might be the main virus antigenic variation sites between sub-clusters “M” and “N” of Den Haag_2006b.

### Structural Modeling the P domains of the NoV GII.4 Sydney_2012 Sub-lineages

**Fig 5:**
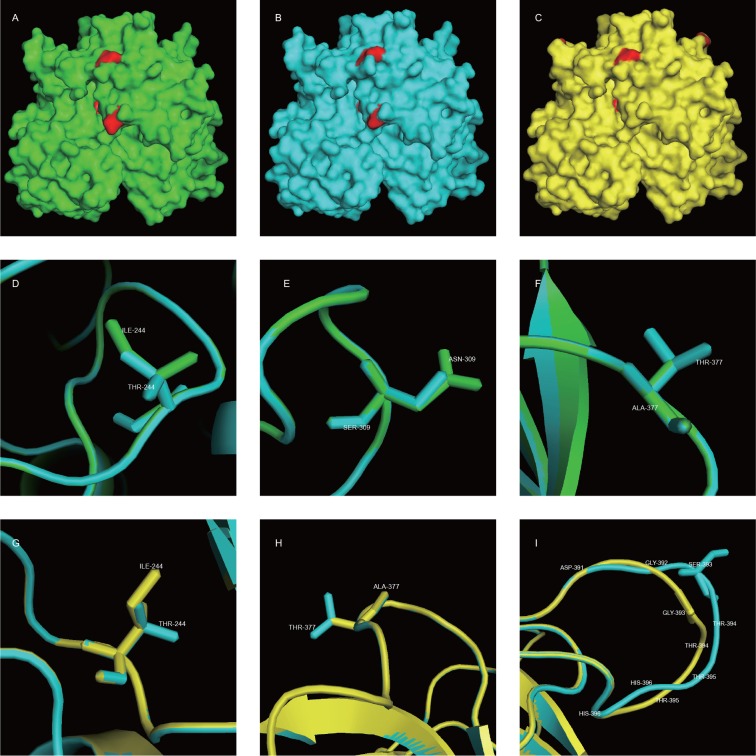
Structure modeling the P domain of the three NoV GII.4 Sydney_2012 sub-lineages. Homology models for the P domain of sub-cluster “O”, sub-cluster “P” and sub-cluster “Q” were generated using the crystal structures of P domain of Norovirus VA387 (PDB accession code: 2OBR) as the template. (A) Surface representation the consensus sequence (GeneBank accession ID: KC473544.1) of sub-cluster “O” P dimer. (B) Surface representation the consensus sequence(GeneBank accession ID: KJ678151.1) of the sub-cluster “P” P dimer. (C) Surface representation the consensus sequence (GeneBank accession ID: KT780378.1) of sub-cluster “Q” P dimer. (D) Cartoon structures representation of the subtle changes (Ile-to-Thr) at position 244 between sub-cluster “O” and sub-cluster “P”. (E) The change from Asn in sub-cluster “O” to Ser in sub-cluster “P” at position 309. (F) A mutation from Ala to Thr at position 377 between sub-cluster “O’ and”P”. (G) Cartoon structures representation of the subtle changes (Thr-to-Ile) at position 244 between sub-cluster “P” and sub-cluster “Q”. (H) The change from Thr in sub-cluster “P” to Ala in sub-cluster “Q” at position 377. (I) A mutation from Ser to Gly at position 393 between sub-cluster “P’ and”Q”.

Likewise, by analyzing 73 protein sequences, amino acid variations (≥5 residues changes) at residues 17, 174, 244, 297, 309, 333, 353, 372, 373, 377, 393, 414, 438 and 539 were identified among sub-cluster "O", "P" and "Q" of GII.4 NoV Sydney_2012 in China (S4 Table). Moreover, homology models for the P domain of Sydney_2012 sub-cluster “O”, “P” and sub-cluster “Q” were also predicted (Fig 5A, 5B and 5C). Three amino acid differences from sub-cluster “O” to sub-cluster “P” at residues 244 (Ile-to-Thr) and 309 (Asn-to-Ser) and 377 (Ala-to-Thr) were observed (Fig 5D, 5E and 5F). In addition, subtle changes Thr-to-Ile at position 244, Thr-to-Ala at position 377 and Ser-to-Gly at position 393 between sub-cluster “P” and sub-cluster “Q” were also discovered (Fig 5G, 5H and 5I).

## Discussion

Norovirus is regarded as the most important causative agent of acute gastroenteritis worldwide. It can spread through contaminated food or water or from person to person and are highly infectious [5]. The majority of NoV infections (70%-80%) have been caused by different GII.4 variants within the last 20 years [4]. Consequently, NoV is a public health problem worldwide, including in China. In this study, to better understand the epidemiological distribution and genetic diversity of these variants circulating in the population of China, NoV GII.4 strains were investigated in 22 Chinese regions between 2004 and 2015.

Structural modeling the P domains of the two representative Den Haag_2006b sub-lineages showed that mutation from Ser393 (sub-cluster “N”) to Gly393 (sub-cluster “M”) predicts this amino acid difference may exert two profound effects upon receptor binding. First, a Ser-to-Gly at position 393 reduces polar potential to epitope D, which may affect some carbohydrate interactions (compare Fig 4C and 4E). Second, the side chain of Gly393 is interact with the adjacent conserved Gly392 and Thr394, resulting in subtle conformation changes of epitope D, which potentially alters carbohydrate binding. The change of 414His (sub-cluster “N”) to 414Pro (sub-cluster “M”) reduces the space in the vicinity of epitope E, and the change at position 255 (Ser-to-Gly) occurs at the outer surface of the P1 domain, adding polar potential to sub-cluster “M”, both of which may slightly influence some carbohydrate interactions. The residue 255 is consistent with the previous Brazilian [35] reports, and the residues 393 and 414 are more responsible for the difference in immunogenicity among Den Haag_2006b via positive selection [19, 34]. As for the NoV GII.4 Sydney_2012 variant, the effects of amino acid mutations including Ile244-to-Thr244, Asn309-to-Ser309, Ala377-to-Thr377 from sub-cluster “O” to sub-cluster “P” and Thr244-to-Ile244, Thr377-to-Ala377 from sub-cluster “P” to sub-cluster “Q” on the three predicted spatial structures were subtle. The mutation Ser393 (sub-cluster “P”) to Gly393 (sub-cluster “Q”) was consistent with Den Haag_2006b lineage. Our results suggest that although these changes are subtle, they may have profound influences on carbohydrate binding affinity between sub-clusters “M” and “N” of Den Haag_2006b and sub-clusters “O”, “P”, and “Q” of Sydney_2012.

Besides, study showed that the epitope D (residues 393–395) can alter histo-blood group antigen (HBGA) binding affinity [36–38], so the antigenic changes that result in escape from herd immunity may also drive changes in HBGA affinities, altering population susceptibility patterns. As suggested by de Rougemont [37], the combination of a high relative affinity for ABH antigens expressed in secretors and the ability to recognize the majority of the non-secretor population may have contributed to the dominance of Den Haag_2006b strain. Further studies including characterization of HBGAs polymorphisms of Chinese population to relate the binding properties of NoV should be conducted for a clear understanding of predominance of variants GII.4 in the country.

In addition, a Bayesian coalescent evolutionary analysis estimated that the NoV GII.4 VP1 capsid gene evolved at a rate of 4.74×10^−3^, 6.99×10^−3^ and 7.68×10^−3^ nucleotide substitutions/site/year for the strict, uncorrelated log-normal model (UCLN) and uncorrelated exponential derivation model (UCED) clocks, respectively (Table 1). This is similar to the rates reported in previous studies for the entire VP1 gene (1.9 to 9.96 substitutions/site/year) [39–42]. A key component in establishing evolutionary mechanisms of the noroviruses is the knowledge of the rate at which noroviruses generate genetic diversity that leads to fixed mutations in the viral population. Given the fact that most RNA viruses evolve at a rate of approximately 1×10^−3^ nucleotide substitutions/site/year [43]. The fast evolution of GII.4 might have allowed the virus to generate replacement clusters and constantly evade the host immune surveillance system within the same genogroup.

Furthermore, the demographic history reconstruction of NoV GII.4 Den Haag_2006b variant demonstrates that the population of the GII.4 Den Haag_2006b variant increased rapidly in late 2005 and reached a maximum effective population size in mid to late 2006, with a relatively constant values until 2010. According to the results obtained in this work, the diversification process of this variant would have started in mid-2005 (time to the most recent common ancestor). Such complex demographic patterns of this variant might be related to the existence of two distinct sub-lineages of Den Haag_2006b. The estimated emergence of the sub-cluster ‘‘M” coincides with the expansion phase of the Den Haag_2006b variant between 2006 and 2007. The spatial and temporal distribution characteristics of sub-cluster ‘‘N” is consistent with stable population distribution between 2007 and 2010 (Figs 2 and 3). Likewise, the demographic history pattern of Sydney_2012 was related to the existence of three distinct sub-clusters. The early sub-cluster “O” is in accordance with the relatively low stable stage during 2012 and 2013, and the later sub-cluster “P” is correspond with the one-year fast expansion phase. The temporal distribution characteristics of sub-cluster ‘‘Q” is consistent with the final stable population distribution between 2014 and 2015 (Figs 2 and 3).

Moreover, the phylogenetic analysis of complete capsid gene (ORF2) sequences allowed the identification of six different variants of NoV GII.4 that were sequentially detected in China over the last decade. Although NoV GII.4 variants were detected in most areas of China, both the number of NoV sequences and the diversity of NoV GII.4 variants were distinct among different regions. In most of the years analyzed, two or three different GII.4 variants were detected simultaneously in China, with a comparable pattern to which observed in Japan (2006–2009) [17], Singapore (2004–2011) [19] and Brazil (2004–2012) [35]. The most prevalent variants were Den Haag_2006b, New Orleans_2009, and Sydney_2012 detected in more than 9 Chinese cities. Most of the GII.4 variants obtained from distinct geographical regions in China appeared to be closely related based on the phylogenetic analysis instead of grouping into distinct lineages according to geographical locations. For example, although the NoV GII.4 Den Haag_2006b variant was found in 19 regions of China, they were clustered into a single branch on the ML and MCC trees (Figs 1 and 2). This suggests that NoV spread and circulate rapidly across China rather than evolving in a region-specific manner.

There are limitations to the result of our analysis. Firstly, the data presented here might not represent the overall molecular epidemiological trend of the 12-year period study due to lack of sampling, particularly during the years 2004 and 2005. Secondly, the Bayesian inference framework requires the researcher first to carry out a Bayesian MCMC analysis of the data, which can be time and memory consuming. Thirdly, as NoV is not a notifiable disease in China, under reporting of NoV outbreaks remains an issue.

In recent years, continuous widespread epidemic of GII.4 NoV created an enormous social and economic burden on communities and health systems. In the present study, the distribution of GII.4 variants over the last decade in China was successively dominated by the pandemic circulation of several variants including Asia_2003, Hunter_2004, Den Haag_2006b, Apeldoorn_2007, New Orleans_2009, and Sydney_2012 continuing to date, which were similar to the global patterns of distribution except few exceptions. We believe that, although our work is limited geographically to China, these data will lead to a better understanding of the epidemiology of this virus and to guide preventive measures against NoV outbreak. To our knowledge, this is the first investigation to conduct temporal dynamic evolutionary analysis of GII.4 variants in China. The relationship between Human NoV infection and disease in China is complex, and many points about its epidemics and evolution remain unknown. Therefore, continual epidemiological surveillance focusing on strain variation and dynamic change is important and necessary for understanding the epidemiology and development of a strategy for disease control and prevention.

## Supporting Information

S1 Fig Phylogenetic Tree of GII.4 NoV variants in China. Phylogenetic relationships of full-length capsid GII.4 strains sequences, constructed by TREE-PUZZLE v5.3 software (174 Chinese sequences and 16 reference sequences, 1623 nt, HKY+I+G4, 10000 bootstraps).
(EPS)

S2 Fig Genetic diversity of GII.4 sequence variants in China from 2004 to 2015. An unrooted tree was generated on the basis of all available region C sequences, constructed by MEGA 6.06 software (526 Chinese sequences, 281 nt, HKY+I+G4).
(EPS)

S3 Fig Temporal distribution of NoV GII.4 variants in China between 2004 and 2015 (n = 624). (EPS)

S1 File Parameter values for the best-fit model of nucleotide substitutions by jModelTest v2.1.10 program. (HTML)

S1 Table Characteristics information of NoV sequences used in our study. (XLS)

S2 Table Geographical distribution of the GII.4 NoV variants detected in China during the period of 2004–2015. (XLS)

S3 Table Amino acid variations sites between sub-cluster "M" and "N" of GII.4 NoV Den_Hagg_2006b in China. A large number of substitutions(≥5 residues changes) at residues 255, 393, 407, 411, 412, 413, 414 and 425 were highlighted. HM802525.1 Hongkong 2006 was choosen as an identical sequence. "." represents that the residue at the position in one strain is the same as amino acid in the corresponding site of the identical sequence.
(XLS)

S4 Table Amino acid variations sites between sub-cluster "O", "P" and "Q" of GII.4 NoV Sydney_2012 in China. A large number of substitutions(≥5 residues changes) at residues 17, 174, 244, 297, 309, 333, 353, 372, 373, 377, 393, 414, 438 and 539 were highlighted. KC473544.1 Huzhou 2012 was choosen as an identical sequence. "." represents that the residue at the position in one strain is the same as amino acid in the corresponding site of the identical sequence.
(XLS)
