# Supplementary material for: Temporal Evolutionary Dynamics of Norovirus GII.4 Variants in China between 2004 and 2015
Source: PLoS One. 2016 Sep 20;11(9):e0163166. doi: 10.1371/journal.pone.0163166 (PMC5029875; doi:10.1371/journal.pone.0163166)
Supplement: S1 File — (HTML) [file pone.0163166.s004.html]

jModelTest Output


# jModeltest 2.1

---

(c) 2011-onwards D. Darriba, G.L. Taboada, R. Doallo and D. Posada,  
(1) Department of Biochemistry, Genetics and Immunology
University of Vigo, 36310 Vigo, Spain.  
(2) Department of Electronics and Systems  
University of A Coruna, 15071 A Coruna, Spain.  
e-mail: ddarriba@udc.es, dposada@uvigo.es  
  
Thu Apr 07 12:34:43 CST 2016   
 Windows 7 6.1, arch: x86, bits: 32, numcores: 8  

|  |  |
| --- | --- |
| Citation: | Darriba D, Taboada GL, Doallo R and Posada D. 2012. "jModelTest 2: more models, new heuristics and parallel computing". Nature Methods 9, 772. |

# Settings

---

Arguments =   
Input Alignment: "C:\Users\admin\Desktop\align\_GII.4\_ORF2\_174.fas"  
NumTaxa = 174  
Length = 1,623  
Phyml version = 3.0  
Phyml binary = PhyML\_3.0\_win32.exe  
Candidate models = 88  
number of substitution schemes = 11  
including models with equal/unequal base frequencies (+F)  
including models with/without a proportion of invariable sites (+I)  
including models with/without rate variation among sites (+G) (nCat = 4)  
Optimized free parameters (K) = Substitution parameters + 345 branch lengths + topology  
Base tree for likelihood calculations = Maximum Likelihood  
Tree topology search operation = NNI  

# Model Optimization Results

---

| ID | Name | Partition | -lnL | p | fA | fC | fG | fT | ti/tv | R(a) | R(b) | R(c) | R(d) | R(e) | R(f) | p-inv | shape |
| --- | --- | --- | --- | --- | --- | --- | --- | --- | --- | --- | --- | --- | --- | --- | --- | --- | --- |
| 1 | JC | 000000 | 13487.3536 | 346 | - | - | - | - | - | - | - | - | - | - | - | - | - |
| 2 | JC+I | 000000 | 12882.3568 | 347 | - | - | - | - | - | - | - | - | - | - | - | 0.6010 | - |
| 3 | JC+G | 000000 | 12801.7787 | 347 | - | - | - | - | - | - | - | - | - | - | - | - | 0.2830 |
| 4 | JC+I+G | 000000 | 12788.6256 | 348 | - | - | - | - | - | - | - | - | - | - | - | 0.4150 | 0.8320 |
| 5 | F81 | 000000 | 13435.8133 | 349 | 0.2089 | 0.3118 | 0.2054 | 0.2739 | - | - | - | - | - | - | - | - | - |
| 6 | F81+I | 000000 | 12818.6944 | 350 | 0.2072 | 0.3144 | 0.1971 | 0.2812 | - | - | - | - | - | - | - | 0.6050 | - |
| 7 | F81+G | 000000 | 12729.4884 | 350 | 0.2013 | 0.3189 | 0.1948 | 0.2850 | - | - | - | - | - | - | - | - | 0.2790 |
| 8 | F81+I+G | 000000 | 12716.6413 | 351 | 0.2008 | 0.3192 | 0.1943 | 0.2857 | - | - | - | - | - | - | - | 0.4140 | 0.7950 |
| 9 | K80 | 010010 | 12385.3416 | 347 | - | - | - | - | 8.6650 | - | - | - | - | - | - | - | - |
| 10 | K80+I | 010010 | 11764.2543 | 348 | - | - | - | - | 9.0078 | - | - | - | - | - | - | 0.6060 | - |
| 11 | K80+G | 010010 | 11678.8272 | 348 | - | - | - | - | 9.1656 | - | - | - | - | - | - | - | 0.2810 |
| 12 | K80+I+G | 010010 | 11661.9426 | 349 | - | - | - | - | 9.1138 | - | - | - | - | - | - | 0.4140 | 0.7940 |
| 13 | HKY | 010010 | 12339.8835 | 350 | 0.2166 | 0.3097 | 0.2042 | 0.2695 | 8.9988 | - | - | - | - | - | - | - | - |
| 14 | HKY+I | 010010 | 11737.5165 | 351 | 0.2340 | 0.2966 | 0.2039 | 0.2655 | 8.9140 | - | - | - | - | - | - | 0.6030 | - |
| 15 | HKY+G | 010010 | 11659.5040 | 351 | 0.2414 | 0.2924 | 0.2044 | 0.2619 | 9.0209 | - | - | - | - | - | - | - | 0.2820 |
| 16 | HKY+I+G | 010010 | 11641.7420 | 352 | 0.2426 | 0.2904 | 0.2048 | 0.2622 | 9.0819 | - | - | - | - | - | - | 0.4080 | 0.8030 |
| 17 | TrNef | 010020 | 12507.5268 | 348 | - | - | - | - | - | 1.0000 | 89.8275 | 1.0000 | 1.0000 | 200.0000 | 1.0000 | - | - |
| 18 | TrNef+I | 010020 | 11915.8612 | 349 | - | - | - | - | - | 1.0000 | 90.9049 | 1.0000 | 1.0000 | 200.0000 | 1.0000 | 0.5820 | - |
| 19 | TrNef+G | 010020 | 11835.5246 | 349 | - | - | - | - | - | 1.0000 | 74.8421 | 1.0000 | 1.0000 | 200.0000 | 1.0000 | - | 0.3000 |
| 20 | TrNef+I+G | 010020 | 11823.9891 | 350 | - | - | - | - | - | 1.0000 | 69.4914 | 1.0000 | 1.0000 | 200.0000 | 1.0000 | 0.4210 | 0.9140 |
| 21 | TrN | 010020 | 12499.9645 | 351 | 0.2701 | 0.2524 | 0.2569 | 0.2207 | - | 1.0000 | 80.7483 | 1.0000 | 1.0000 | 200.0000 | 1.0000 | - | - |
| 22 | TrN+I | 010020 | 11913.1582 | 352 | 0.2783 | 0.2466 | 0.2490 | 0.2260 | - | 1.0000 | 82.4968 | 1.0000 | 1.0000 | 200.0000 | 1.0000 | 0.5810 | - |
| 23 | TrN+G | 010020 | 11828.3662 | 352 | 0.2802 | 0.2483 | 0.2465 | 0.2250 | - | 1.0000 | 69.3665 | 1.0000 | 1.0000 | 200.0000 | 1.0000 | - | 0.3010 |
| 24 | TrN+I+G | 010020 | 11808.1362 | 353 | 0.2808 | 0.2465 | 0.2466 | 0.2261 | - | 1.0000 | 63.6610 | 1.0000 | 1.0000 | 200.0000 | 1.0000 | 0.4210 | 0.9160 |
| 25 | TPM1 | 012210 | 12470.3154 | 348 | - | - | - | - | - | 1.0000 | 200.0000 | 13.6700 | 13.6700 | 200.0000 | 1.0000 | - | - |
| 26 | TPM1+I | 012210 | 11836.5177 | 349 | - | - | - | - | - | 1.0000 | 200.0000 | 14.7892 | 14.7892 | 200.0000 | 1.0000 | 0.6070 | - |
| 27 | TPM1+G | 012210 | 11753.4054 | 349 | - | - | - | - | - | 1.0000 | 200.0000 | 13.9227 | 13.9227 | 200.0000 | 1.0000 | - | 0.2740 |
| 28 | TPM1+I+G | 012210 | 11742.6019 | 350 | - | - | - | - | - | 1.0000 | 200.0000 | 14.0941 | 14.0941 | 200.0000 | 1.0000 | 0.4220 | 0.8170 |
| 29 | TPM1uf | 012210 | 12424.4536 | 351 | 0.2176 | 0.3093 | 0.2041 | 0.2689 | - | 1.0000 | 200.0000 | 13.8078 | 13.8078 | 200.0000 | 1.0000 | - | - |
| 30 | TPM1uf+I | 012210 | 11809.5764 | 352 | 0.2361 | 0.2942 | 0.2037 | 0.2660 | - | 1.0000 | 200.0000 | 14.8615 | 14.8615 | 200.0000 | 1.0000 | 0.6010 | - |
| 31 | TPM1uf+G | 012210 | 11731.8968 | 352 | 0.2443 | 0.2916 | 0.2044 | 0.2598 | - | 1.0000 | 200.0000 | 14.3842 | 14.3842 | 200.0000 | 1.0000 | - | 0.2780 |
| 32 | TPM1uf+I+G | 012210 | 11718.9764 | 353 | 0.2439 | 0.2903 | 0.2052 | 0.2607 | - | 1.0000 | 200.0000 | 14.3990 | 14.3990 | 200.0000 | 1.0000 | 0.4190 | 0.8250 |
| 33 | TPM2 | 010212 | 12433.0680 | 348 | - | - | - | - | - | 15.3234 | 200.0000 | 15.3234 | 1.0000 | 200.0000 | 1.0000 | - | - |
| 34 | TPM2+I | 010212 | 11804.5573 | 349 | - | - | - | - | - | 14.8785 | 200.0000 | 14.8785 | 1.0000 | 200.0000 | 1.0000 | 0.6060 | - |
| 35 | TPM2+G | 010212 | 11720.4635 | 349 | - | - | - | - | - | 14.7378 | 200.0000 | 14.7378 | 1.0000 | 200.0000 | 1.0000 | - | 0.2780 |
| 36 | TPM2+I+G | 010212 | 11703.1251 | 350 | - | - | - | - | - | 14.9054 | 200.0000 | 14.9054 | 1.0000 | 200.0000 | 1.0000 | 0.4130 | 0.7850 |
| 37 | TPM2uf | 010212 | 12385.4895 | 351 | 0.2117 | 0.3088 | 0.2110 | 0.2684 | - | 14.9750 | 200.0000 | 14.9750 | 1.0000 | 200.0000 | 1.0000 | - | - |
| 38 | TPM2uf+I | 010212 | 11780.5585 | 352 | 0.2270 | 0.2939 | 0.2142 | 0.2649 | - | 14.9628 | 200.0000 | 14.9628 | 1.0000 | 200.0000 | 1.0000 | 0.5990 | - |
| 39 | TPM2uf+G | 010212 | 11705.2758 | 352 | 0.2298 | 0.2896 | 0.2199 | 0.2607 | - | 14.8205 | 200.0000 | 14.8205 | 1.0000 | 200.0000 | 1.0000 | - | 0.2820 |
| 40 | TPM2uf+I+G | 010212 | 11690.7282 | 353 | 0.2297 | 0.2879 | 0.2209 | 0.2615 | - | 14.8823 | 200.0000 | 14.8823 | 1.0000 | 200.0000 | 1.0000 | 0.4220 | 0.8470 |
| 41 | TPM3 | 012012 | 12523.6322 | 348 | - | - | - | - | - | 10.3025 | 200.0000 | 1.0000 | 10.3025 | 200.0000 | 1.0000 | - | - |
| 42 | TPM3+I | 012012 | 11888.3533 | 349 | - | - | - | - | - | 11.5515 | 200.0000 | 1.0000 | 11.5515 | 200.0000 | 1.0000 | 0.6060 | - |
| 43 | TPM3+G | 012012 | 11796.7591 | 349 | - | - | - | - | - | 11.5212 | 200.0000 | 1.0000 | 11.5212 | 200.0000 | 1.0000 | - | 0.2770 |
| 44 | TPM3+I+G | 012012 | 11781.3664 | 350 | - | - | - | - | - | 11.5878 | 200.0000 | 1.0000 | 11.5878 | 200.0000 | 1.0000 | 0.4110 | 0.7630 |
| 45 | TPM3uf | 012012 | 12482.3151 | 351 | 0.2175 | 0.3032 | 0.2036 | 0.2756 | - | 10.1115 | 200.0000 | 1.0000 | 10.1115 | 200.0000 | 1.0000 | - | - |
| 46 | TPM3uf+I | 012012 | 11859.9795 | 352 | 0.2367 | 0.2890 | 0.2022 | 0.2722 | - | 11.6868 | 200.0000 | 1.0000 | 11.6868 | 200.0000 | 1.0000 | 0.5990 | - |
| 47 | TPM3uf+G | 012012 | 11779.7593 | 352 | 0.2455 | 0.2805 | 0.2032 | 0.2709 | - | 11.6054 | 200.0000 | 1.0000 | 11.6054 | 200.0000 | 1.0000 | - | 0.2780 |
| 48 | TPM3uf+I+G | 012012 | 11764.9073 | 353 | 0.2456 | 0.2783 | 0.2034 | 0.2727 | - | 11.7296 | 200.0000 | 1.0000 | 11.7296 | 200.0000 | 1.0000 | 0.4180 | 0.8090 |
| 49 | TIM1ef | 012230 | 12468.0974 | 349 | - | - | - | - | - | 1.0000 | 200.0000 | 13.9374 | 13.9374 | 200.0000 | 1.0000 | - | - |
| 50 | TIM1ef+I | 012230 | 11836.1307 | 350 | - | - | - | - | - | 1.0000 | 200.0000 | 14.7843 | 14.7843 | 200.0000 | 1.0000 | 0.6080 | - |
| 51 | TIM1ef+G | 012230 | 11751.9100 | 350 | - | - | - | - | - | 1.0000 | 200.0000 | 13.8859 | 13.8859 | 200.0000 | 1.0000 | - | 0.2750 |
| 52 | TIM1ef+I+G | 012230 | 11747.7208 | 351 | - | - | - | - | - | 1.0000 | 200.0000 | 14.0879 | 14.0879 | 200.0000 | 1.0000 | 0.4290 | 0.8420 |
| 53 | TIM1 | 012230 | 12426.9866 | 352 | 0.2179 | 0.3078 | 0.2043 | 0.2701 | - | 1.0000 | 200.0000 | 14.0559 | 14.0559 | 200.0000 | 1.0000 | - | - |
| 54 | TIM1+I | 012230 | 11808.9274 | 353 | 0.2368 | 0.2949 | 0.2033 | 0.2650 | - | 1.0000 | 200.0000 | 14.9269 | 14.9269 | 200.0000 | 1.0000 | 0.6030 | - |
| 55 | TIM1+G | 012230 | 11732.0963 | 353 | 0.2451 | 0.2916 | 0.2040 | 0.2594 | - | 1.0000 | 200.0000 | 14.3732 | 14.3732 | 200.0000 | 1.0000 | - | 0.2780 |
| 56 | TIM1+I+G | 012230 | 11718.9401 | 354 | 0.2439 | 0.2902 | 0.2051 | 0.2608 | - | 1.0000 | 200.0000 | 14.3985 | 14.3985 | 200.0000 | 1.0000 | 0.4230 | 0.8420 |
| 57 | TIM2ef | 010232 | 12433.8247 | 349 | - | - | - | - | - | 15.0541 | 200.0000 | 15.0541 | 1.0000 | 200.0000 | 1.0000 | - | - |
| 58 | TIM2ef+I | 010232 | 11806.9108 | 350 | - | - | - | - | - | 14.7352 | 200.0000 | 14.7352 | 1.0000 | 200.0000 | 1.0000 | 0.6010 | - |
| 59 | TIM2ef+G | 010232 | 11720.3836 | 350 | - | - | - | - | - | 14.7413 | 200.0000 | 14.7413 | 1.0000 | 200.0000 | 1.0000 | - | 0.2770 |
| 60 | TIM2ef+I+G | 010232 | 11712.5075 | 351 | - | - | - | - | - | 14.9507 | 200.0000 | 14.9507 | 1.0000 | 200.0000 | 1.0000 | 0.4160 | 0.7940 |
| 61 | TIM2 | 010232 | 12385.4926 | 352 | 0.2117 | 0.3088 | 0.2111 | 0.2684 | - | 14.9755 | 200.0000 | 14.9755 | 1.0000 | 200.0000 | 1.0000 | - | - |
| 62 | TIM2+I | 010232 | 11783.9127 | 353 | 0.2270 | 0.2931 | 0.2143 | 0.2656 | - | 14.6331 | 200.0000 | 14.6331 | 1.0000 | 200.0000 | 1.0000 | 0.6040 | - |
| 63 | TIM2+G | 010232 | 11705.9858 | 353 | 0.2309 | 0.2903 | 0.2192 | 0.2596 | - | 14.9101 | 200.0000 | 14.9101 | 1.0000 | 200.0000 | 1.0000 | - | 0.2810 |
| 64 | TIM2+I+G | 010232 | 11690.7282 | 354 | 0.2297 | 0.2879 | 0.2209 | 0.2615 | - | 14.8823 | 200.0000 | 14.8823 | 1.0000 | 200.0000 | 1.0000 | 0.4220 | 0.8470 |
| 65 | TIM3ef | 012032 | 12521.0344 | 349 | - | - | - | - | - | 10.3078 | 200.0000 | 1.0000 | 10.3078 | 200.0000 | 1.0000 | - | - |
| 66 | TIM3ef+I | 012032 | 11888.6752 | 350 | - | - | - | - | - | 11.2460 | 200.0000 | 1.0000 | 11.2460 | 200.0000 | 1.0000 | 0.6070 | - |
| 67 | TIM3ef+G | 012032 | 11798.4538 | 350 | - | - | - | - | - | 11.3622 | 200.0000 | 1.0000 | 11.3622 | 200.0000 | 1.0000 | - | 0.2760 |
| 68 | TIM3ef+I+G | 012032 | 11782.5403 | 351 | - | - | - | - | - | 11.4310 | 200.0000 | 1.0000 | 11.4310 | 200.0000 | 1.0000 | 0.4130 | 0.7690 |
| 69 | TIM3 | 012032 | 12477.0207 | 352 | 0.2176 | 0.3034 | 0.2040 | 0.2751 | - | 10.1113 | 200.0000 | 1.0000 | 10.1113 | 200.0000 | 1.0000 | - | - |
| 70 | TIM3+I | 012032 | 11860.2265 | 353 | 0.2377 | 0.2887 | 0.2011 | 0.2724 | - | 11.6773 | 200.0000 | 1.0000 | 11.6773 | 200.0000 | 1.0000 | 0.6000 | - |
| 71 | TIM3+G | 012032 | 11779.0396 | 353 | 0.2454 | 0.2809 | 0.2032 | 0.2706 | - | 11.7546 | 200.0000 | 1.0000 | 11.7546 | 200.0000 | 1.0000 | - | 0.2800 |
| 72 | TIM3+I+G | 012032 | 11764.8747 | 354 | 0.2449 | 0.2782 | 0.2041 | 0.2728 | - | 11.7480 | 200.0000 | 1.0000 | 11.7480 | 200.0000 | 1.0000 | 0.4170 | 0.8070 |
| 73 | TVMef | 012314 | 12406.2500 | 350 | - | - | - | - | - | 12.2257 | 200.0000 | 17.6004 | 7.5495 | 200.0000 | 1.0000 | - | - |
| 74 | TVMef+I | 012314 | 11779.1759 | 351 | - | - | - | - | - | 11.3844 | 200.0000 | 17.0773 | 8.3127 | 200.0000 | 1.0000 | 0.6010 | - |
| 75 | TVMef+G | 012314 | 11695.6853 | 351 | - | - | - | - | - | 11.8689 | 200.0000 | 16.6117 | 7.9283 | 200.0000 | 1.0000 | - | 0.2780 |
| 76 | TVMef+I+G | 012314 | 11680.2588 | 352 | - | - | - | - | - | 11.7065 | 200.0000 | 16.4439 | 7.8959 | 200.0000 | 1.0000 | 0.4460 | 0.9040 |
| 77 | TVM | 012314 | 12381.0565 | 353 | 0.2113 | 0.3063 | 0.2081 | 0.2743 | - | 19.1241 | 200.0000 | 11.6941 | 4.9858 | 200.0000 | 1.0000 | - | - |
| 78 | TVM+I | 012314 | 11758.5183 | 354 | 0.2282 | 0.2954 | 0.2103 | 0.2662 | - | 10.9754 | 200.0000 | 17.3831 | 8.9622 | 200.0000 | 1.0000 | 0.5990 | - |
| 79 | TVM+G | 012314 | 11941.8141 | 354 | 0.2324 | 0.2835 | 0.2141 | 0.2700 | - | 30.7698 | 200.0000 | 0.0100 | 0.0100 | 200.0000 | 1.0000 | - | 0.2730 |
| 80 | TVM+I+G | 012314 | 11663.2463 | 355 | 0.2329 | 0.2905 | 0.2152 | 0.2613 | - | 11.5963 | 200.0000 | 17.1959 | 8.4227 | 200.0000 | 1.0000 | 0.4560 | 1.0030 |
| 81 | SYM | 012345 | 12321.3437 | 351 | - | - | - | - | - | 9.2480 | 101.0032 | 13.1297 | 5.7659 | 200.0000 | 1.0000 | - | - |
| 82 | SYM+I | 012345 | 11779.4168 | 352 | - | - | - | - | - | 11.3906 | 200.0000 | 17.0839 | 8.3189 | 200.0000 | 1.0000 | 0.6040 | - |
| 83 | SYM+G | 012345 | 11683.3516 | 352 | - | - | - | - | - | 9.0727 | 103.8701 | 12.4740 | 6.5740 | 200.0000 | 1.0000 | - | 0.2930 |
| 84 | SYM+I+G | 012345 | 11649.8998 | 353 | - | - | - | - | - | 9.0900 | 99.3412 | 12.1451 | 6.5216 | 200.0000 | 1.0000 | 0.4270 | 0.9390 |
| 85 | GTR | 012345 | 12403.6535 | 354 | 0.2144 | 0.3097 | 0.2089 | 0.2670 | - | 11.3533 | 200.0000 | 21.3100 | 10.1653 | 200.0000 | 0.0100 | - | - |
| 86 | GTR+I | 012345 | 11735.9393 | 355 | 0.2491 | 0.2685 | 0.2391 | 0.2432 | - | 8.8835 | 114.4724 | 13.6460 | 6.8858 | 200.0000 | 1.0000 | 0.5920 | - |
| 87 | GTR+G | 012345 | 11644.1922 | 355 | 0.2517 | 0.2762 | 0.2312 | 0.2409 | - | 1.2525 | 16.9732 | 1.8800 | 0.8954 | 28.6514 | 1.0000 | - | 0.2910 |
| 88 | GTR+I+G | 012345 | 11646.6255 | 356 | 0.2507 | 0.2668 | 0.2390 | 0.2435 | - | 8.4337 | 104.5875 | 12.6963 | 6.6123 | 200.0000 | 1.0000 | 0.4180 | 0.8970 |

# AIC Selection Results

---

## Model selected

|  |  |  |
| --- | --- | --- |
| Model | HKY+I+G | |
| partition | 010010 | |
| -lnL | 11641.7420 | |
| K | 352 | |
| freqA | 0.2426 | R(a) | - |
| freqC | 0.2904 | R(b) | - |
| freqG | 0.2048 | R(c) | - |
| freqT | 0.2622 | R(d) | - |
| ti/tv | 9.0819 | R(e) | - |
|  |  | R(f) | - |
| p-inv | 0.4080 | gamma | 0.8030 |

# AIC Results

---

| Model | -lnL | K | AIC | delta | weight | cumWeight |
| --- | --- | --- | --- | --- | --- | --- |
| HKY+I+G | 11641.7420 | 352 | 23987.4840 | 0.0000 | 0.9955 | 0.9955 |
| GTR+G | 11644.1922 | 355 | 23998.3845 | 10.9004 | 0.0043 | 0.9998 |
| GTR+I+G | 11646.6255 | 356 | 24005.2509 | 17.7669 | 0.0001 | 0.9999 |
| SYM+I+G | 11649.8998 | 353 | 24005.7995 | 18.3155 | 0.0001 | 1.0000 |
| HKY+G | 11659.5040 | 351 | 24021.0079 | 33.5239 | 0.0000 | 1.0000 |
| K80+I+G | 11661.9426 | 349 | 24021.8852 | 34.4012 | 0.0000 | 1.0000 |
| TVM+I+G | 11663.2463 | 355 | 24036.4926 | 49.0086 | 0.0000 | 1.0000 |
| K80+G | 11678.8272 | 348 | 24053.6544 | 66.1704 | 0.0000 | 1.0000 |
| TVMef+I+G | 11680.2588 | 352 | 24064.5176 | 77.0336 | 0.0000 | 1.0000 |
| SYM+G | 11683.3516 | 352 | 24070.7031 | 83.2191 | 0.0000 | 1.0000 |
| TPM2uf+I+G | 11690.7282 | 353 | 24087.4564 | 99.9724 | 0.0000 | 1.0000 |
| TIM2+I+G | 11690.7282 | 354 | 24089.4564 | 101.9724 | 0.0000 | 1.0000 |
| TVMef+G | 11695.6853 | 351 | 24093.3706 | 105.8866 | 0.0000 | 1.0000 |
| TPM2+I+G | 11703.1251 | 350 | 24106.2501 | 118.7661 | 0.0000 | 1.0000 |
| TPM2uf+G | 11705.2758 | 352 | 24114.5515 | 127.0675 | 0.0000 | 1.0000 |
| TIM2+G | 11705.9858 | 353 | 24117.9716 | 130.4876 | 0.0000 | 1.0000 |
| TIM2ef+I+G | 11712.5075 | 351 | 24127.0150 | 139.5309 | 0.0000 | 1.0000 |
| TPM2+G | 11720.4635 | 349 | 24138.9271 | 151.4430 | 0.0000 | 1.0000 |
| TIM2ef+G | 11720.3836 | 350 | 24140.7672 | 153.2831 | 0.0000 | 1.0000 |
| TPM1uf+I+G | 11718.9764 | 353 | 24143.9528 | 156.4688 | 0.0000 | 1.0000 |
| TIM1+I+G | 11718.9401 | 354 | 24145.8803 | 158.3962 | 0.0000 | 1.0000 |
| TPM1uf+G | 11731.8968 | 352 | 24167.7935 | 180.3095 | 0.0000 | 1.0000 |
| TIM1+G | 11732.0963 | 353 | 24170.1926 | 182.7086 | 0.0000 | 1.0000 |
| HKY+I | 11737.5165 | 351 | 24177.0329 | 189.5489 | 0.0000 | 1.0000 |
| GTR+I | 11735.9393 | 355 | 24181.8785 | 194.3945 | 0.0000 | 1.0000 |
| TPM1+I+G | 11742.6019 | 350 | 24185.2039 | 197.7198 | 0.0000 | 1.0000 |
| TIM1ef+I+G | 11747.7208 | 351 | 24197.4415 | 209.9575 | 0.0000 | 1.0000 |
| TIM1ef+G | 11751.9100 | 350 | 24203.8200 | 216.3359 | 0.0000 | 1.0000 |
| TPM1+G | 11753.4054 | 349 | 24204.8108 | 217.3268 | 0.0000 | 1.0000 |
| K80+I | 11764.2543 | 348 | 24224.5087 | 237.0246 | 0.0000 | 1.0000 |
| TVM+I | 11758.5183 | 354 | 24225.0367 | 237.5526 | 0.0000 | 1.0000 |
| TPM3uf+I+G | 11764.9073 | 353 | 24235.8147 | 248.3306 | 0.0000 | 1.0000 |
| TIM3+I+G | 11764.8747 | 354 | 24237.7494 | 250.2654 | 0.0000 | 1.0000 |
| TVMef+I | 11779.1759 | 351 | 24260.3519 | 272.8678 | 0.0000 | 1.0000 |
| TPM3+I+G | 11781.3664 | 350 | 24262.7327 | 275.2487 | 0.0000 | 1.0000 |
| SYM+I | 11779.4168 | 352 | 24262.8335 | 275.3495 | 0.0000 | 1.0000 |
| TPM3uf+G | 11779.7593 | 352 | 24263.5186 | 276.0346 | 0.0000 | 1.0000 |
| TIM3+G | 11779.0396 | 353 | 24264.0792 | 276.5952 | 0.0000 | 1.0000 |
| TPM2uf+I | 11780.5585 | 352 | 24265.1169 | 277.6329 | 0.0000 | 1.0000 |
| TIM3ef+I+G | 11782.5403 | 351 | 24267.0806 | 279.5966 | 0.0000 | 1.0000 |
| TIM2+I | 11783.9127 | 353 | 24273.8253 | 286.3413 | 0.0000 | 1.0000 |
| TPM3+G | 11796.7591 | 349 | 24291.5182 | 304.0341 | 0.0000 | 1.0000 |
| TIM3ef+G | 11798.4538 | 350 | 24296.9077 | 309.4236 | 0.0000 | 1.0000 |
| TPM2+I | 11804.5573 | 349 | 24307.1146 | 319.6306 | 0.0000 | 1.0000 |
| TIM2ef+I | 11806.9108 | 350 | 24313.8216 | 326.3376 | 0.0000 | 1.0000 |
| TrN+I+G | 11808.1362 | 353 | 24322.2724 | 334.7883 | 0.0000 | 1.0000 |
| TPM1uf+I | 11809.5764 | 352 | 24323.1528 | 335.6688 | 0.0000 | 1.0000 |
| TIM1+I | 11808.9274 | 353 | 24323.8548 | 336.3708 | 0.0000 | 1.0000 |
| TrNef+I+G | 11823.9891 | 350 | 24347.9782 | 360.4941 | 0.0000 | 1.0000 |
| TrN+G | 11828.3662 | 352 | 24360.7324 | 373.2484 | 0.0000 | 1.0000 |
| TrNef+G | 11835.5246 | 349 | 24369.0491 | 381.5651 | 0.0000 | 1.0000 |
| TPM1+I | 11836.5177 | 349 | 24371.0354 | 383.5514 | 0.0000 | 1.0000 |
| TIM1ef+I | 11836.1307 | 350 | 24372.2615 | 384.7774 | 0.0000 | 1.0000 |
| TPM3uf+I | 11859.9795 | 352 | 24423.9590 | 436.4749 | 0.0000 | 1.0000 |
| TIM3+I | 11860.2265 | 353 | 24426.4529 | 438.9689 | 0.0000 | 1.0000 |
| TPM3+I | 11888.3533 | 349 | 24474.7066 | 487.2226 | 0.0000 | 1.0000 |
| TIM3ef+I | 11888.6752 | 350 | 24477.3504 | 489.8664 | 0.0000 | 1.0000 |
| TrNef+I | 11915.8612 | 349 | 24529.7223 | 542.2383 | 0.0000 | 1.0000 |
| TrN+I | 11913.1582 | 352 | 24530.3165 | 542.8324 | 0.0000 | 1.0000 |
| TVM+G | 11941.8141 | 354 | 24591.6282 | 604.1441 | 0.0000 | 1.0000 |
| SYM | 12321.3437 | 351 | 25344.6874 | 1357.2033 | 0.0000 | 1.0000 |
| HKY | 12339.8835 | 350 | 25379.7669 | 1392.2829 | 0.0000 | 1.0000 |
| K80 | 12385.3416 | 347 | 25464.6832 | 1477.1992 | 0.0000 | 1.0000 |
| TVM | 12381.0565 | 353 | 25468.1130 | 1480.6289 | 0.0000 | 1.0000 |
| TPM2uf | 12385.4895 | 351 | 25472.9790 | 1485.4950 | 0.0000 | 1.0000 |
| TIM2 | 12385.4926 | 352 | 25474.9851 | 1487.5011 | 0.0000 | 1.0000 |
| TVMef | 12406.2500 | 350 | 25512.4999 | 1525.0159 | 0.0000 | 1.0000 |
| GTR | 12403.6535 | 354 | 25515.3070 | 1527.8230 | 0.0000 | 1.0000 |
| TPM1uf | 12424.4536 | 351 | 25550.9072 | 1563.4231 | 0.0000 | 1.0000 |
| TIM1 | 12426.9866 | 352 | 25557.9732 | 1570.4892 | 0.0000 | 1.0000 |
| TPM2 | 12433.0680 | 348 | 25562.1360 | 1574.6519 | 0.0000 | 1.0000 |
| TIM2ef | 12433.8247 | 349 | 25565.6494 | 1578.1654 | 0.0000 | 1.0000 |
| TIM1ef | 12468.0974 | 349 | 25634.1948 | 1646.7108 | 0.0000 | 1.0000 |
| TPM1 | 12470.3154 | 348 | 25636.6308 | 1649.1468 | 0.0000 | 1.0000 |
| TIM3 | 12477.0207 | 352 | 25658.0415 | 1670.5574 | 0.0000 | 1.0000 |
| TPM3uf | 12482.3151 | 351 | 25666.6303 | 1679.1462 | 0.0000 | 1.0000 |
| TrN | 12499.9645 | 351 | 25701.9290 | 1714.4449 | 0.0000 | 1.0000 |
| TrNef | 12507.5268 | 348 | 25711.0536 | 1723.5696 | 0.0000 | 1.0000 |
| TIM3ef | 12521.0344 | 349 | 25740.0689 | 1752.5848 | 0.0000 | 1.0000 |
| TPM3 | 12523.6322 | 348 | 25743.2644 | 1755.7804 | 0.0000 | 1.0000 |
| F81+I+G | 12716.6413 | 351 | 26135.2827 | 2147.7986 | 0.0000 | 1.0000 |
| F81+G | 12729.4884 | 350 | 26158.9769 | 2171.4928 | 0.0000 | 1.0000 |
| JC+I+G | 12788.6256 | 348 | 26273.2512 | 2285.7671 | 0.0000 | 1.0000 |
| JC+G | 12801.7787 | 347 | 26297.5573 | 2310.0733 | 0.0000 | 1.0000 |
| F81+I | 12818.6944 | 350 | 26337.3889 | 2349.9048 | 0.0000 | 1.0000 |
| JC+I | 12882.3568 | 347 | 26458.7136 | 2471.2296 | 0.0000 | 1.0000 |
| F81 | 13435.8133 | 349 | 27569.6266 | 3582.1425 | 0.0000 | 1.0000 |
| JC | 13487.3536 | 346 | 27666.7071 | 3679.2231 | 0.0000 | 1.0000 |

---

|  |  |
| --- | --- |
| -lnL: | negative log likelihod |
| K: | number of estimated parameters |
| AIC: | Akaike Information Criterion |
| delta: | AIC difference |
| weight: | AIC weight |
| cumWeight: | cumulative AIC weight |

jModelTest 2.1. D.Darriba, G.L.Taboada, R.Doallo and D.Posada
